# Visualization of Zika Virus Infection via a Light-Initiated Bio-Orthogonal Cycloaddition Labeling Strategy

**DOI:** 10.3389/fbioe.2022.940511

**Published:** 2022-07-08

**Authors:** Judun Zheng, Rui Yue, Ronghua Yang, Qikang Wu, Yunxia Wu, Mingxing Huang, Xu Chen, Weiqiang Lin, Jialin Huang, Xiaodong Chen, Yideng Jiang, Bin Yang, Yuhui Liao

**Affiliations:** ^1^ Molecular Diagnosis and Treatment Center for Infectious Diseases, Dermatology Hospital, Southern Medical University, Guangzhou, China; ^2^ Department of Burn and Plastic Surgery, Guangzhou First People’s Hospital, South China University of Technology, Guangzhou, China; ^3^ Department of Clinical Laboratory, The First People’s Hospital of Foshan, Foshan, China; ^4^ Department of Burn Surgery & Department of Rheumatology, The First People’s Hospital of Foshan, Foshan, China; ^5^ Department of Infectious Disease, the Fifth Affiliated Hospital, Sun Yat-sen University, Zhuhai, China; ^6^ NHC Key Laboratory of Metabolic Cardiovascular Diseases Research, Ningxia Key Laboratory of Vascular Injury and Repair Research, Ningxia Medical University, Yinchuan, China

**Keywords:** Zika virus, quantum dots (DQs), light-initiated cycloaddition, fluorescent probe, phenanthrenequinone

## Abstract

Zika virus (ZIKV) is a re-emerging flavivirus that leads to devastating consequences for fetal development. It is crucial to visualize the pathogenicity activities of ZIKV ranging from infection pathways to immunity processes, but the accurate labeling of ZIKV remains challenging due to the lack of a reliable labeling technique. We introduce the photo-activated bio-orthogonal cycloaddition to construct a fluorogenic probe for the labeling and visualizing of ZIKV. Via a simple UV photoirradiation, the fluorogenic probes could be effectively labeled on the ZIKV. We demonstrated that it can be used for investigating the interaction between ZIKV and diverse cells and avoiding the autofluorescence phenomenon in traditional immunofluorescence assay. Thus, this bioorthogonal-enabled labeling strategy can serve as a promising approach to monitor and understand the interaction between the ZIKV and host cells.

## 1 Introduction

The mosquito-transmitted Zika virus (ZIKV), which belongs to the family Flaviviridae and genus flavivirus, can cause several Zika syndrome including ventriculomegaly and microcephaly (Miner and Diamond, 2017). Recent outbreaks of ZIKV have been reported in more than 30 countries or territories, emerging as a major threat to global health (Sampathkumar and Sanchez, 2016; Hills et al., 2017; Mengesha Tsegaye et al., 2018). The current diagnostic techniques available for ZIKV rely on the reverse transcriptase-polymerase chain reaction assay or ZIKV-specific IgM antibody testing, which play an important role in preventing the spread of disease (Ergünay et al., 2010; Barreiro, 2016; Kikuti et al., 2018; Low et al., 2021). The exploration of cellular signaling pathways on ZIKV infection has also attracted considerable research attention, greatly enriching the study of mechanisms of infection of RNA viruses (Grant et al., 2016; Chen et al., 2018; Chiramel and Best, 2018; Garcia et al., 2020). Among these, visualization of the Zika virus-host cell interactions is essential to comprehend the molecular mechanisms and pathogenesis of ZIKV disease.

Fluorescent dyes have been widely used in viral labeling and real-time imaging, which improve our understanding of the viral infection process (Zheng et al., 2019; Liao et al., 2020; Zhang et al., 2020). Thus, the photobleaching and spectral overlaps of fluorophores are inescapable, greatly affecting the efficacy of fluorophores, greatly affecting the efficacy of tracking dye-labeled viruses, and limiting the application in bioimaging. Fluorescent quantum dots (QDs) can be rationally chosen as an alternative candidate because they have distinguishing optical properties including narrow-band and tunable fluorescence emission, high fluorescence quantum yields, and photostability (Matea et al., 2017; Pleskova et al., 2018). Currently, numerous efforts have been devoted to constructing the QDs–virus imaging modality and demonstrating its capabilities in providing meaningful information (You et al., 2006; Zhang et al., 2012; Ribeiro et al., 2019; Kuang et al., 2020; Chen et al., 2021; Chung and Zhang, 2021; Lin et al., 2021; He et al., 2022; Yi et al., 2022). It is crucial to reveal the real virus–host cell interaction transversion by maintaining viral infiltration after riveting the virus on QDs (Cui et al., 2011; Liu et al., 2012; Hong et al., 2015; Ma et al., 2017). However, riveting virus on QDs via the unmild and uncontrollable physical-chemical process remains challenging.

**SCHEME 1:**
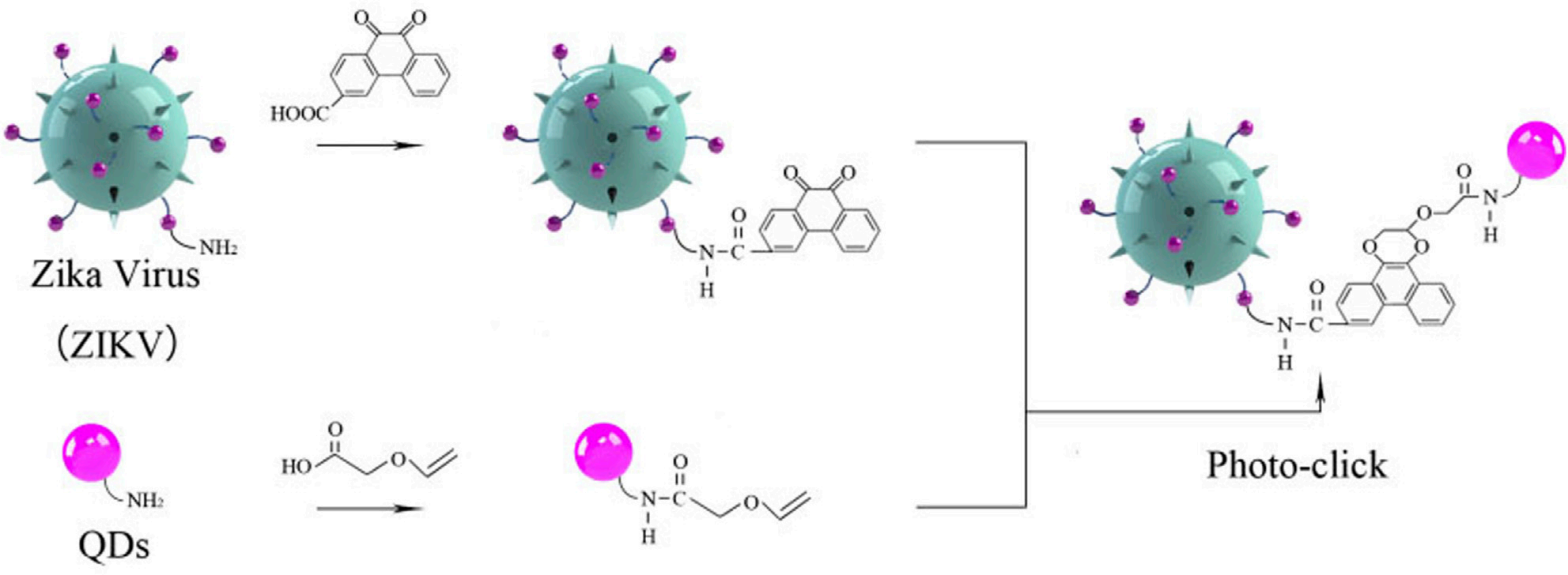
Schematic illustration of Zika virus labeling and imaging via light-initiated photo-click cycloaddition.

To address this, we rationally designed a novel strategy with the photo-click cycloaddition-based QDs to tag and track the ZIKV (Scheme1). The light-initiated bio-orthogonal photo-click reaction has been widely applied in numerous biolabeling and bioimaging, enabling visualization of specific biomolecules with precise spatiotemporal control in their native environment (Lim and Lin, 2011; Huang et al., 2013; Herner and Lin, 2016). With this strategy, ZIKV was successfully tracked and visualized after cell entry in different cell lines, such as A549 and SNB19. Moreover, the ZIKV-QDs can map the ZIKV–host cell interactions under chlorpromazine hydrochloride (CPZ)- or nocodazole-treated conditions. This strategy would provide a reliable toolbox to elucidate the virus–host cell interactions and develop potential rapid diagnosis and therapeutic approaches.

## 2 Experimental Section

### 2.1 Materials and Reagents

Syto13 was obtained from Sigma. Amino-labeled QDs were obtained from Wuhan Jiayuan Quantum Dots Co., Ltd (Wuhan, China). The cell counting kit-8 (CCK-8) was obtained from Dojindo Laboratories (Kumamoto, Japan). 1-ethyl-3-(3-dimethylaminopropyl) carbodiimide (EDCI), N-hydroxysuccinimid esters (NHS), and N,N-4-dimethylaminopyridine (DMAP) were purchased from Energy Chemical (Shanghai, China).

### 2.2 Cell Lines

A549 cell and SNB19 cell were purchased from ATCC. The cells were cultured in Dulbecco’s modified Eagle medium (DMEM) (Gibco, Ltd., Grand Island, NY, USA). The media were supplemented with 10% FBS (Gibco), 50 U mL^−1^ penicillin, and 50 μg ml^−1^ streptomycin. The cells were maintained in a humidified 37°C incubator in 5% CO_2_.

### 2.3 Modification of Zika Virus

9,10-phenanthrenequinone (PQ, 11 mg, 4.17*10^–5^ mol) was dispersed into 10 ml MES buffer, EDC (80 mg, 4.17*10^–4^ mol) and NHS (120 mg, 4.17*10^–4^ mol) were added, and then ultrasonic was applied for 15 s, the sealing film was sealed, and then it was shaken at 37°C for 15 min. Then, 100 µl of Zika virus (concentration is 2 mg/ml) was dispersed in 10 ml PBS. The liquid was added to the aforementioned MES buffer, shaken at 37°C overnight, and then purified by a NAP-5 desalting column to obtain Zika virus modified with the PQ group.

### 2.4 Modification of Quantum Dots

Take 1 µl of quantum dots (QDs) with the carboxyl group (molar concentration is 8*10^–6^), vinyl ether (VE) 1,000 μl, EDCI 15.3 mg, DAMP 9.8 mg, and put them into a 3-neck flask, respectively, then add 5 ml of dichloromethane to completely dissolve. Then, shake for 2 h at room temperature in a dark environment. Then, go through silica gel column chromatography to obtain quantum dots modified with vinyl ether group.

### 2.5 The Click Reaction of Zika Virus and Quantum Dots

The Zika viruses and the quantum dots were first modified with the PQ group and vinyl ether group, respectively. After that, these modified ZIKV and QDs were dissolved in a PBS solution and then irradiated with the LED lamp for 1 min to obtain the Zika virus modified with the quantum dots (ZIKV-QDs).

### 2.6 ZIKV-QD Internalization Assays

ZIKV-QDs and viral nucleic acid dye Syto13 were mixed and incubated for 1 h and then ultracentrifuged at 10,000 g for 0.5 h to remove the remaining dye. Then mixed with Vero or cells and shook 5 times with an interval of 15 min and then incubated in 5% carbon dioxide at 37°C for 24 h. Then, the cells were fixed with 4% paraformaldehyde, and the fluorescence imaging was observed under the confocal laser scanning microscope (CLSM).

### 2.7 Cell Culture and Cytotoxicity Evaluation *In Vitro*

SNB19 cells were cultured in DMEM containing 10% fetal bovine serum and 1% penicillin/streptomycin at 37°C in a humidified 5% CO_2_ atmosphere. Cell density was determined using a hemocytometer before experimentation. Relative cell viabilities were determined by the standard CCK-8 assay. SNB19 cells were seeded into 96-well plates (10^4^ cells per well). After cells were cultured for 12 h, they were added to a fresh culture medium and excited with a LED lamp (0.5 W/cm^2^) at different times. After incubation at 37°C for 12 h, those cells were added containing 10% CCK-8 DMEM (100 µl). After incubation for 2 h at 37°C, OD_450_, the absorbance value at 450 nm, was measured with a microplate reader to determine the cell viability.

### 2.8 Confocal Laser Scanning Microscope

The aforementioned fluorescent dye particles were photographed with a confocal laser scanning microscope (LSM880). The excitation wavelength of the quantum dots is 561 nm, and the emission wavelength is 605 nm. The excitation wavelength of Syto13 is 488 nm, and the emission wavelength is 509 nm.

## 3 Results and Discussion

### 3.1 Feasibility and Characterizations of QD-Modified Zika Virus

**FIGURE 1:**
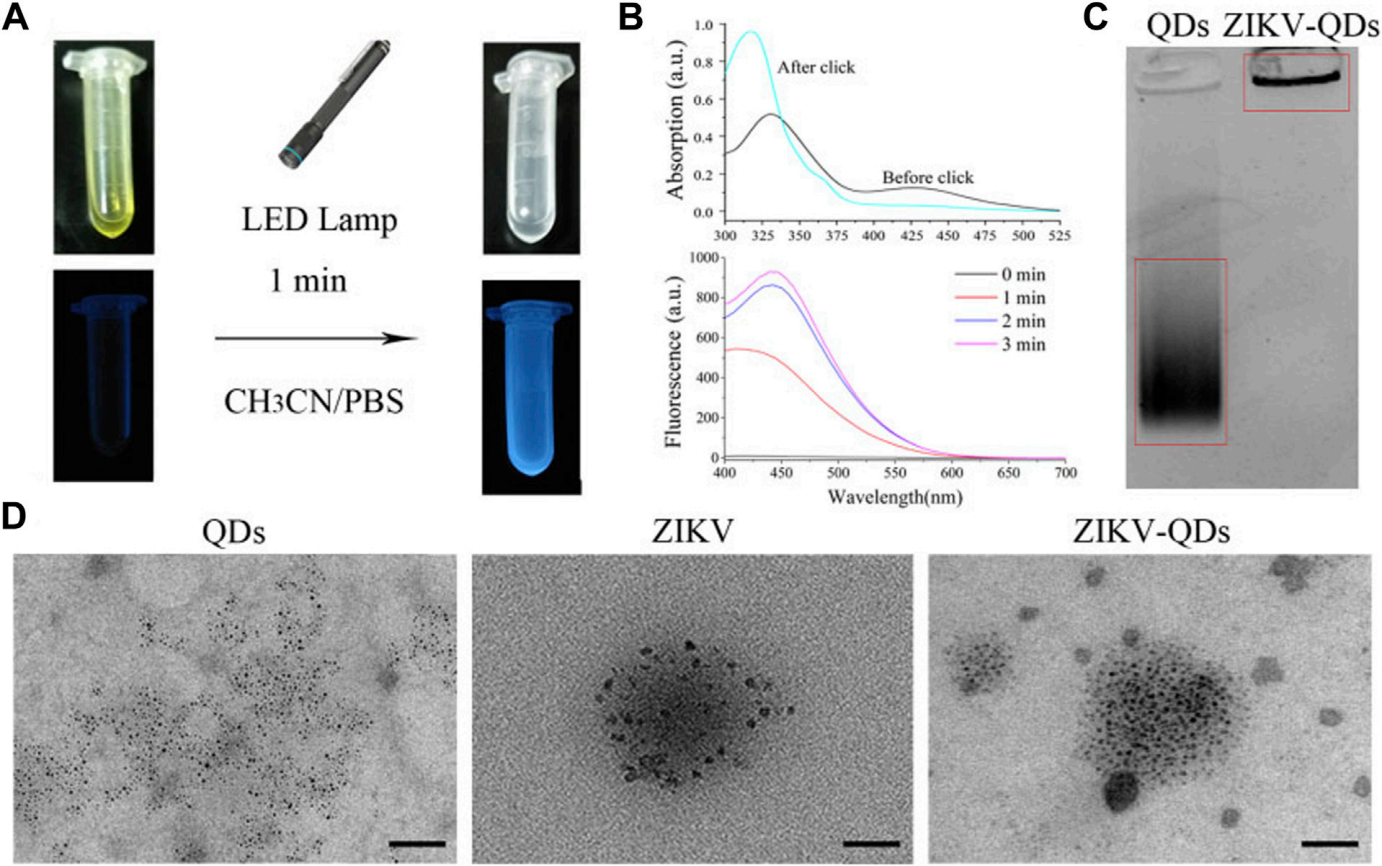
Characterizations of Zika virus labeling and imaging via light-initiated bio-orthogonal photo-click cycloaddition. **(A)** Images of ZIKV and QD solution without (left) or with (right) the irradiation of the LED lamp. **(B)** Absorbance and fluorescence changes of ZIKV and QDs in CH3CN/PBS solution upon irradiation treatment. **(C)** Polyacrylamide gel electrophoresis analysis of QDs and ZIKV-QDs. **(D)** Transmission electron microscopy (TEM) of QDs, ZIKV, and ZIKV-QDs.

Photo-click reaction has been developed as an alternative strategy for spatiotemporally labeling and imaging (Lim and Lin, 2011; Li et al., 2018). Among them, 9,10-phenanthrenequinone (PQ) and electron-rich vinyl ether were widely chosen as photo-click substrates (Supplementary Figure S1, S2). As previous reports revealed that the ZIKV could be chemically labeled with the chemical proteomics strategies owning to the ZIKV surface E proteins to understand the early-stage entry of ZIKV into host cells (Srivastava et al., 2020; King and Irigoyen, 2021). To prepare a fluorescent tracking method, PQ and vinyl ether were functionalized in the ZIKV and QDs, respectively. Then, the ZIKV was labeled with fluorescent QDs via the cyclization under the irradiation (Scheme 1). As shown in Figure 1A, the primary absorbance peak of the as-prepared 9,10-phenanthrenequinone exhibited a significant blue shift that goes from 330 to 310 nm (Supplementary Figure S3), and the absorbance band at 425 nm disappeared along with the irradiation of LED lamp, which was in accordance with the 9,10-phenanthrenequinone analog reported by Zhang group (Li et al., 2018).

Accordingly, the fluorescence intensity of PQ reacted with electron-rich vinyl ether at 450 nm was found to be greatly increased upon increasing irradiation time or concentration (Supplementary Figure S4), resulting in the photo-click reaction between PQ and electron-rich vinyl ether (Coelho et al., 2017). Meanwhile, the color of their mixed solution changed from yellow to colorless, and the blue light can be found, which was consistent with the antecedent results of absorbance and fluorescence (Figure 1B). In addition, the polyacrylamide gel electrophoresis analysis was chosen to validate whether the QDs were labeled on the ZIKV. As shown in Figure 1C, ZIKV-QDs held the original position compared to the QDs, which contributed to the macromolecular proteins of viruses conjugated with QDs by photo-click cycloaddition reaction. On the other hand, transmission electron microscopy (TEM) of ZIKV-QDs shows the typical ZIKV pattern functionalized with QDs, intuitively verifying that the QDs have been successfully decorated in the ZIKV (Figure 1D). Taken together, the photo-click cyclization between PQ and vinyl ether can be applied to label the ZIKV with QDs.

### 3.2 Quantitative Evaluation of QD-Modified Zika Virus

It is crucial to obtain high-purity viruses in various fields, ranging from virus pathogenesis to structure decomposition and vaccine development. Herein, the ZIKV particles were purified using a discontinuous sucrose gradient (Petruska et al., 2002), and all fractions were titrated by plaque assay. We observed the presence of ZIKV in all fractions, and the purified fraction has high titers above 10^8^ PFU/ml (Supplementary Figure S5). In addition, a high RNA level was obtained at a sucrose concentration of 20%, resulting in 5.0 × 10^10^ RNA copies/ml (Supplementary Figure S6). To characterize the modification of QDs on ZIKV, we analyzed the particle sizes of ZIKV and ZIKV-QDs using the NanoSight NS300 equipment. As shown in Supplementary Figures S7 and S8, the average size increased from 108.9 ± 8.2 nm to 119.3 ± 11.5 nm, indicating that the QDs were successfully modified on ZIKV. Furthermore, the zeta potential data showed that the complex of ZIKV-QDs has a higher negative potential (−15.47 mV) than that of the individual QDs (−8.14 mV) and ZIKV (−12.03 mV) (Supplementary Figure S9). This result also indicated the successful modification of QDs on ZIKV through the amido bond between the carboxyl group and amino group. Additionally, no significant difference between the ZIKV and ZIKV-QDs viral titers was observed (Supplementary Figure S10), suggesting that the labeling process does not affect the infiltration performance.

### 3.3 Evaluation of Infiltration Capability With QD-Modified Zika Virus

**FIGURE 2:**
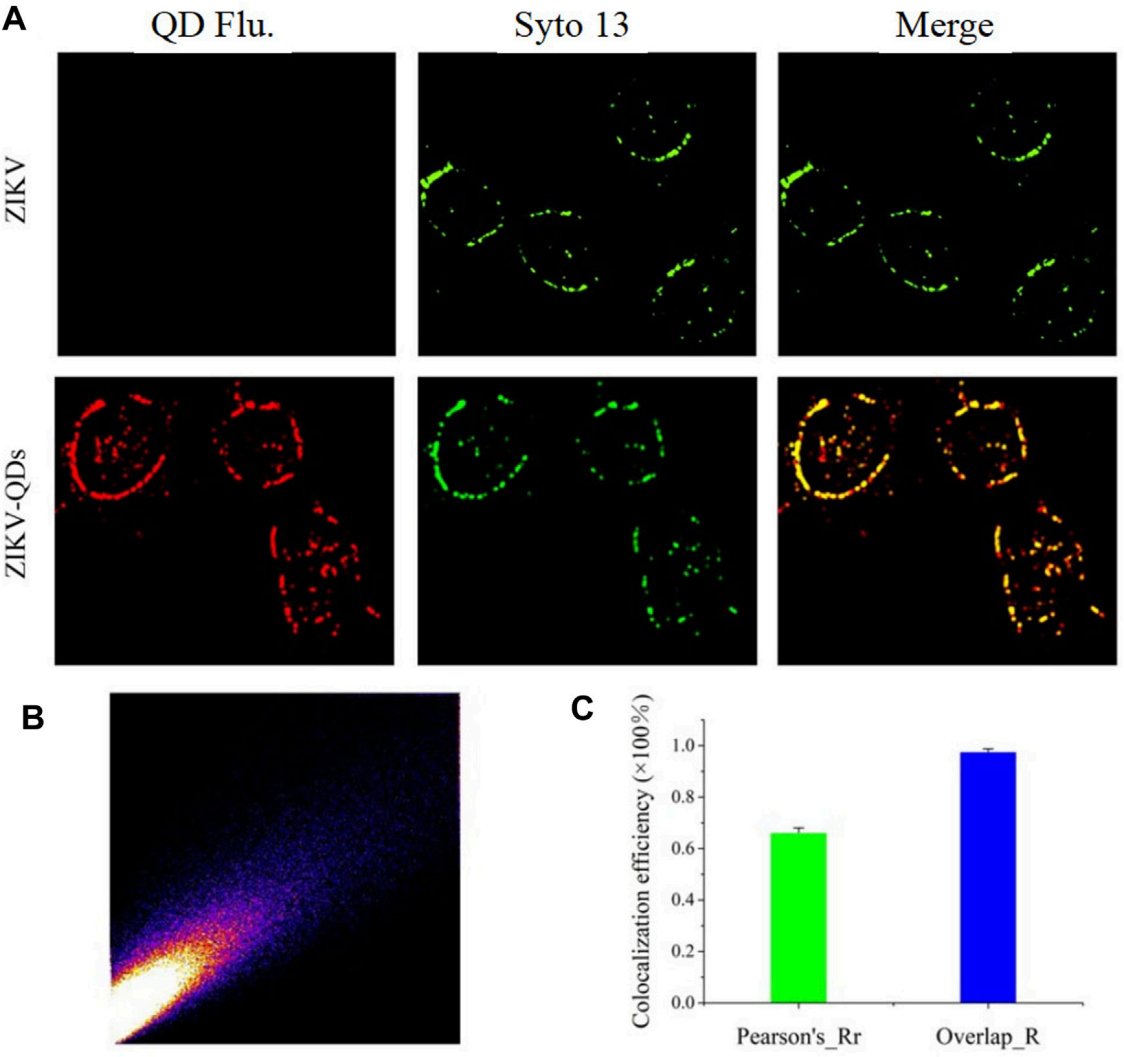
**(A)** Confocal microscopic images of ZIKV nucleic acid colocalized with the commercially available organelle trackers Syto13 in SNB19 cells (Flu is the abbreviation for fluorescence). **(B)** Colocalization scatterplots of **(A)**. **(C)** Corresponding Pearson correlation coefficient of **(A)**.

Next, we evaluated its infiltration capability using the QD-modified ZIKV. In comparison absence of evidence for ZIKV infection in cells, we thus applied QD-modified ZIKV in mapping the infection process. The fluorescence imaging of A549 cells incubated with ZIKV-QDs in the red channel was found to be much brighter compared to the ZIKV group without QDs, and no significant difference in the green emission of Syto13 dye was observed under both conditions (Supplementary Figure S11A). As expected, the fluorescence image of QDs overlaps well with that of a commercially available fluorescent dye of the nucleic acid tracking dye Syto13. Also, the corresponding Mander’s overlap coefficient, a well-established colocation parameter, was performed as 0.94 for the ZIKV-QDs group (Supplementary Figure S11B, S11C). In order to confirm that the QDs were labeled on the ZIKV, ZIKV-QDs were first incubated with the nucleic acid dye Syto13. After that, Syto13-pretreated ZIKV-QDs mixed with cells and subsequently observed interaction behavior between ZIKV and host cell under the confocal laser scanning microscope. To verify the wide application of Syto13-pretreated ZIKV-QDs targeted tracing performance, we also obtained significant fluorescence colocalization experiments on the SNB19 cancer cell line via confocal fluorescence microscopy (Figure 2A). The Pearson correlation coefficient had been drawn via the confocal image (Figure 2B), and the corresponding Mander’s overlap coefficient had also been calculated to be 0.97 (Figure 2C). These bioimaging results demonstrated that the ZIKV labeled with QDs could localize and trace the functions in virus infection procedures.

### 3.4 Evaluation of Drug Efficacy Through the QD-Modified Zika Virus

**FIGURE 3:**
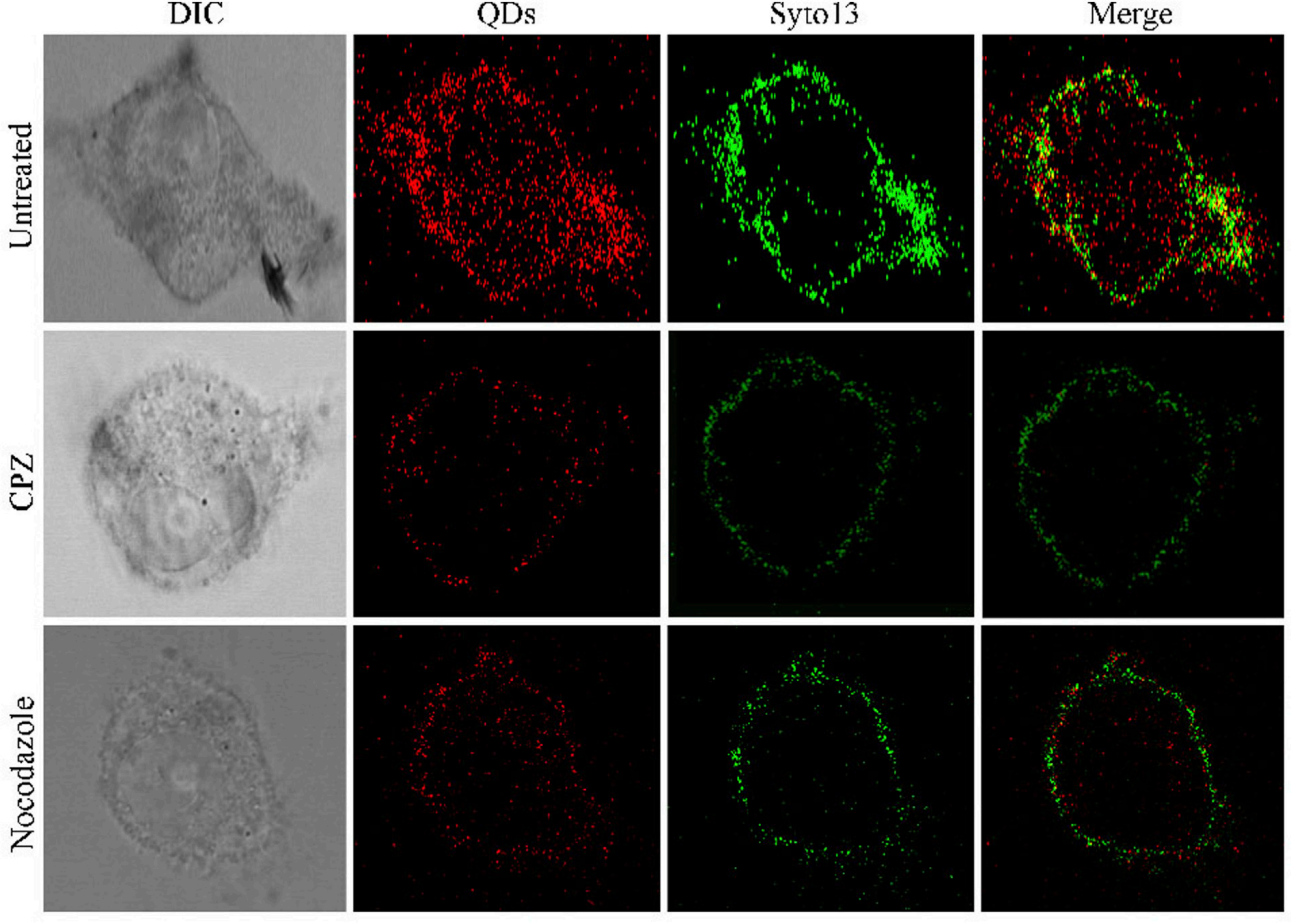
Confocal microscopic images of ZIKV nucleic acid colocalized with the commercially available organelle trackers Syto13 in SNB19 cells treated with different drugs including chlorpromazine and nocodazole.

Furthermore, we demonstrated its applicability for assessing the efficacy of ZIKV-related treatments. Here, we introduced two typical kinds of antivirus drugs including CPZ and nocodazole, which could specifically inhibit clathrin-dependent endocytosis and formation of the microtubule, respectively (Xia and Liang, 2019; Sathyamoorthy et al., 2020). The cellular fluorescence intensity of QDs in the group treated with CPZ decreased compared to the control group (Figure 3). This was attributed to the less ZIKV crossing the monolayer of the barrier of Vero cells. In addition, the treatment of nocodazole also obviously abated the entry and infection of ZIKV at 2 h post-inoculation, providing direct evidence that the microtubule polymerization is required for intracellular trafficking of the ZIKV. These results prompt us to use the fluorescent nanoprobe QDs to trace the process of ZIKV infection and evaluate the treatment efficacy with the independent fluorescent signals.

**FIGURE 4:**
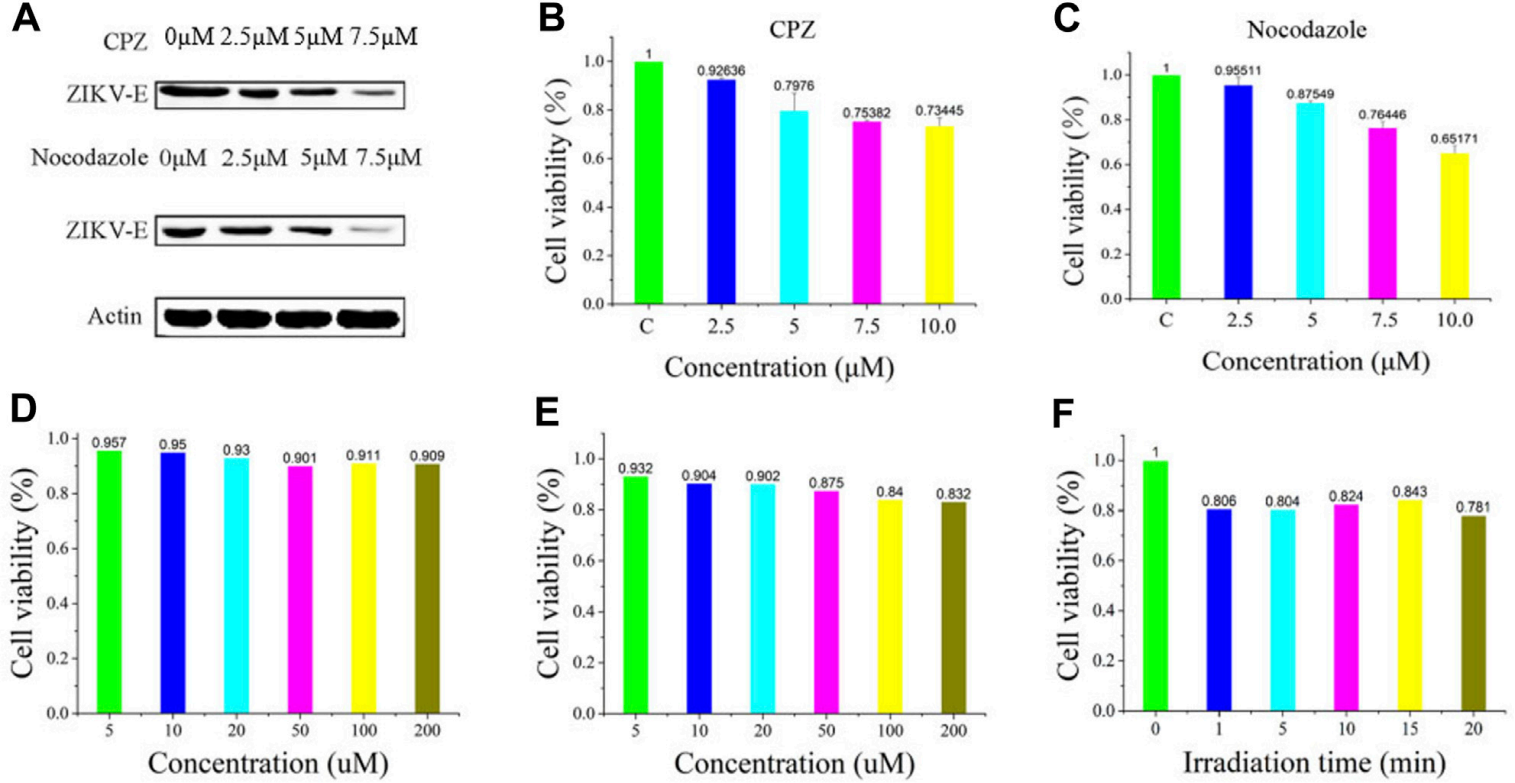
**(A)** Levels of β-actin and ZIKV envelope protein E (ZIKV E) of SNB19 cells treated with CPZ or nocodazole. Cytotoxicity studies of **(B)** CPZ and **(C)** nocodazole. Cytotoxicity studies of **(D)** PQ and **(E)** VE, or **(F)** irradiation time.

To further validate the infiltration activity of the QD-labeled ZIKV, we chose the level of nascent protein synthesis to evaluate the ZIKV infection after being treated with CPZ or nocodazole. ZIKV envelope protein E (ZIKV E) is the major structural protein exposed on the cell surface of the particle, which is engaged in viral attachment, penetration, and membrane fusion. Thus, we chose the ZIKV E to study and evaluate the reason why hydrochloride and nocodazole could induce the infection of ZIKV. Western blot experiments indicate that ZIKV infection had a dramatic effect on the synthesis of ZIKV E (Figure 4A, top), similar to the case that cells infected with the translation inhibitor nocodazole (Figure 4A, bottom). At the same time, the CPZ and nocodazole exhibited some side effects (Figures 4B, C), which means these two drugs could not only inhibit the microtubule polymerization but also lead to the cellular apoptosis due to the disruption of intracellular trafficking (Liao et al., 2018). Furthermore, to test the biological application ability of pre-QDs, the cytotoxicity of PQ and VE was tested with Vero cells. After incubation with different concentrations of PQ and VE, there was no significant change in SNB19 cells (Figures 4D, E). In order to evaluate the influence of light on cells, the effect of light was investigated by the standard CCK-8 assay. Following incubation of various times of light ranging from 0 to 5 min, the cellular viabilities were determined to exceed 80% (Figure 4F), indicating its low effect with the LED lamp laser. Taken together, these aforementioned findings indicated that ZIKV-QDs could accurately map and localize the ZIKV via monitoring the fluorescence of QDs under various conditions.

## 4 Conclusion

In this work, we developed a novel QD-based probe for reliable labeling and visualization of the Zika virus. This probe leverages photo-activated bio-orthogonal cycloaddition for high-efficient conjugation of ZIKV and QDs, exhibiting a simple labeling process and no influence on the infiltration performance. We verified that such a conjugation of ZIKV and QD probe can localize and trace the functions in the virus infection procedure. Moreover, the infiltration activity of the QD-labeled ZIKV is validated using the anti-ZIKV drugs including CPZ or nocodazole by monitoring the level of nascent protein synthesis. These findings suggest that the proposed QD probe could accurately map and localize the ZIKV via monitoring the fluorescence. Thus, this bioorthogonal-enabled QD probe might be a promising approach for monitoring the pathogenicity activities of ZIKV.

## Data Availability

The datasets presented in this study can be found in online repositories. The names of the repository/repositories and accession number(s) can be found in the article/Supplementary Material.
