# Supplementary material for: Visualization of Zika Virus Infection via a Light-Initiated Bio-Orthogonal Cycloaddition Labeling Strategy
Source: Front Bioeng Biotechnol. 2022 Jul 8;10:940511. doi: 10.3389/fbioe.2022.940511 (PMC9305201; doi:10.3389/fbioe.2022.940511)
Supplement: Supplementary file 1 [file DataSheet1.docx]

Supplementary Material

Visualization of Zika Virus Infection via Light-Initiated Bio-orthogonal Cycloaddition Labeling Strategy

**Judun Zheng^1‡^, Rui Yue^1‡^, Ronghua Yang^2‡^, Qikang Wu^3‡^, Yunxia Wu^3^, Mingxing Huang^4^, Xu Chen^4^, Weiqiang Lin^1^, Jialin Huang^1^, Xiaodong Chen^3*^, Yideng Jiang^5*^, Bin Yang^1*^ and Yuhui Liao^1, 4, 5*^**

^1^Molecular Diagnosis and Treatment Center for Infectious Diseases, Dermatology Hospital, Southern Medical University, Guangzhou 510091, China.

^2^Department of Burn and Plastic Surgery, Guangzhou First People's Hospital, South China University of Technology, Guangzhou, Guangdong, China.

^3^Department of Clinical Laboratory, Department of Burn Surgery & Department of Rheumatology, the First People’s Hospital of Foshan, Foshan 528000, China.

^4^Department of Infectious Disease, the Fifth Affiliated Hospital, Sun Yat-sen University, Zhuhai 519000, Guangdong, China.

^5^NHC Key Laboratory of Metabolic Cardiovascular Diseases Research, Ningxia Key Laboratory of Vascular Injury and Repair Research, Ningxia Medical University, Yinchuan, 750004, China.

‡ These authors contributed equally.

*** Correspondence:**

Xiaodong Chen, Email: cxd234@163.com

Yideng Jiang, Email:jwcjyd@163.com

Bin Yang, Email: yangbin1@smu.edu.cn

Yuhui Liao, Email: liaoyh8@mail.sysu.edu.cn

Keywords: Zika virus, Quantum dots, Light-initiated cycloaddition, Fluorescent probe, Phenanthrenequinone.


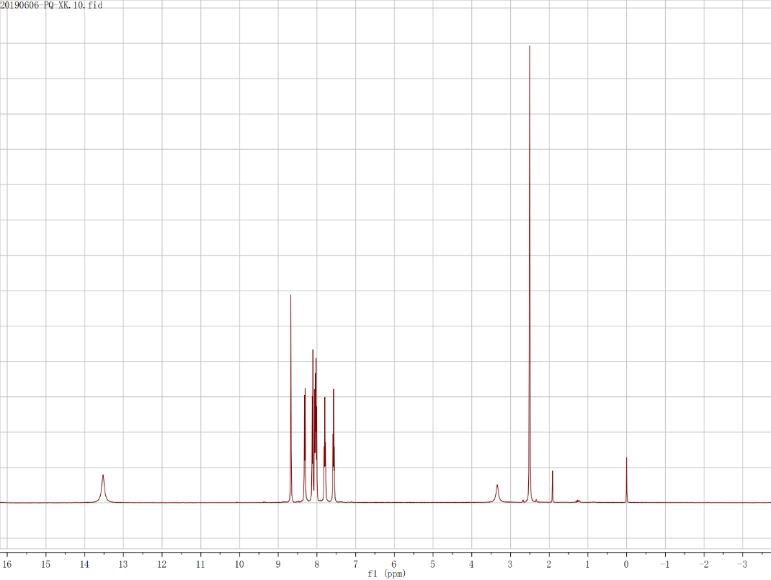


Figure S1. The ^1^H spectrum of 9,10-phenanthrenequinone.


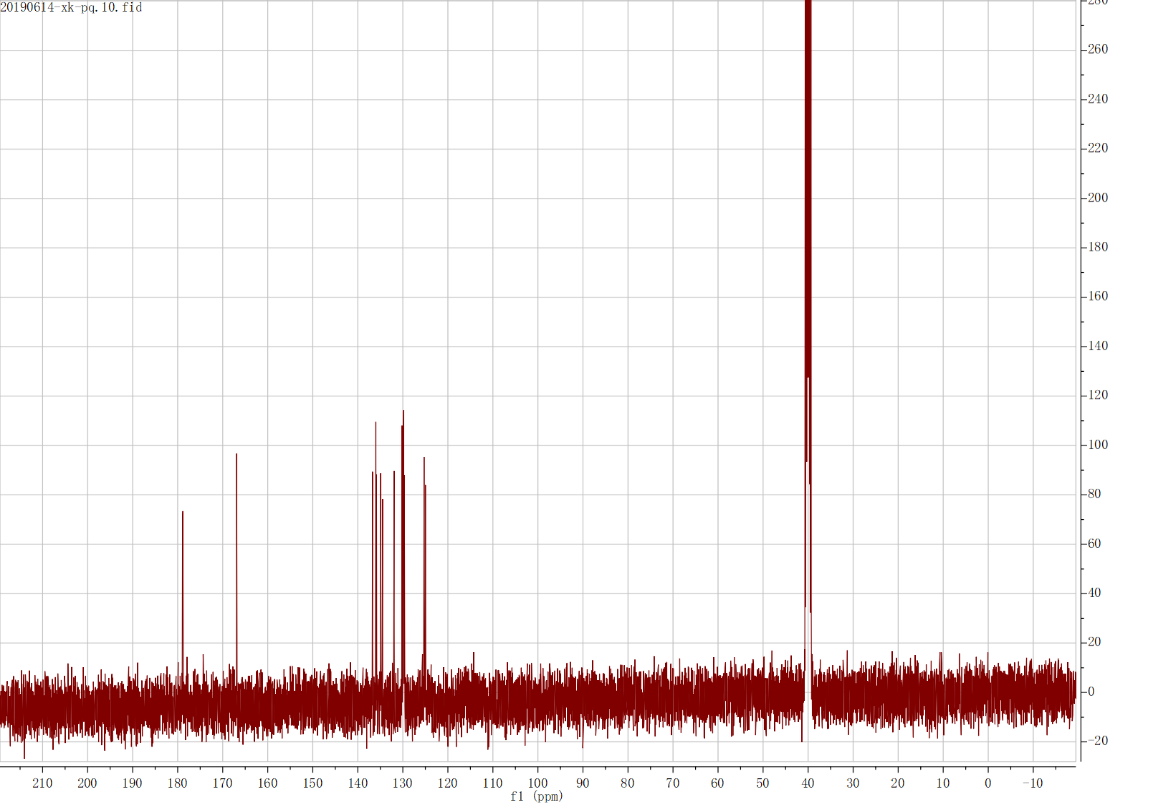


Figure S2. The ^13^C spectrum of 9,10-phenanthrenequinone.





Figure S3. The absorption spectrum of 9,10-phenanthrenequinone.


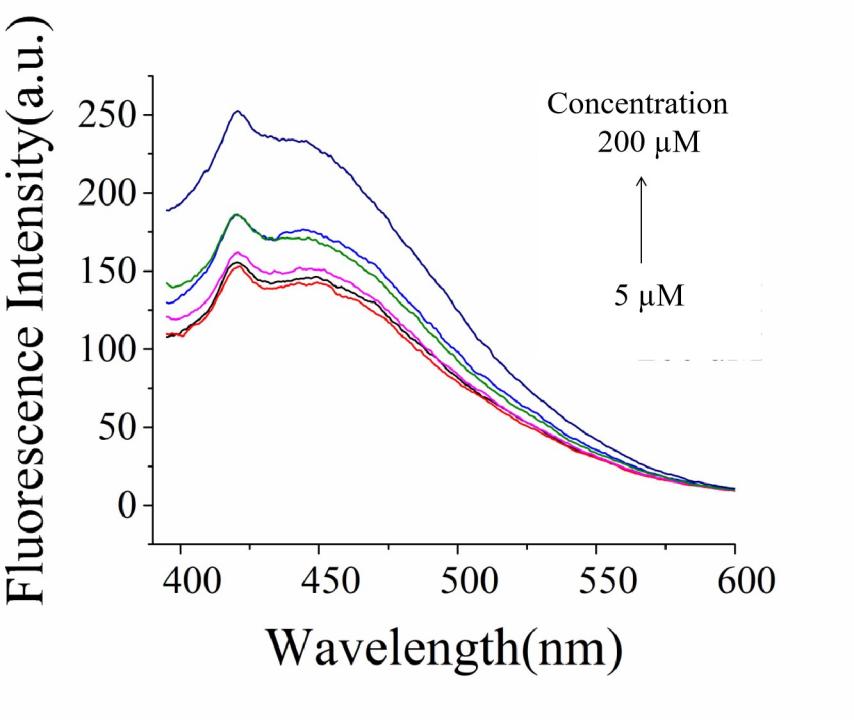


Figure S4. The fluorescence of 9,10-phenanthrenequinone.


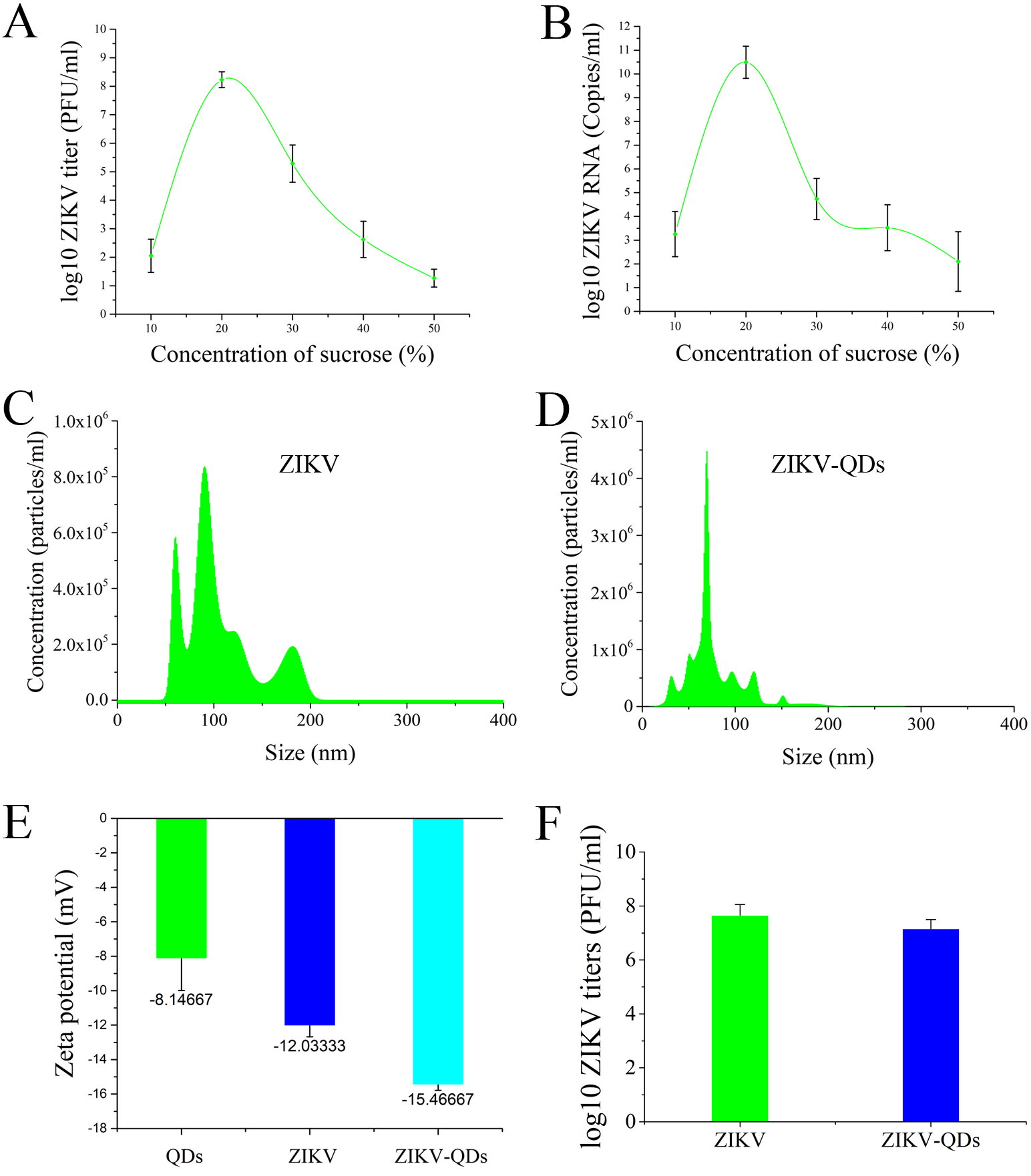


Figure S5. The titers counting of the purified fraction of ZIKV virus.


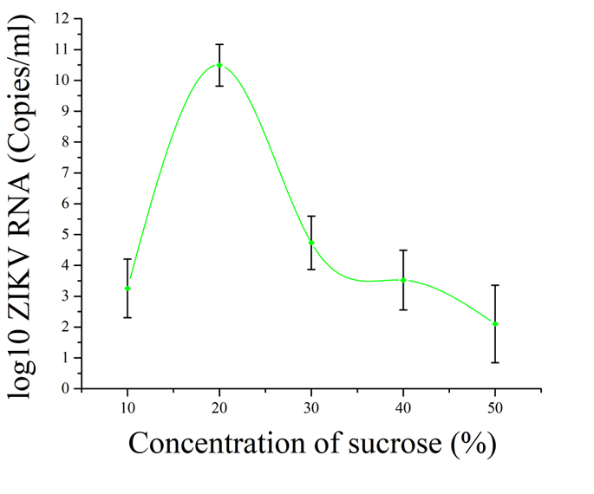


Figure S6. The ZIKV RNA level with a sucrose concentration.


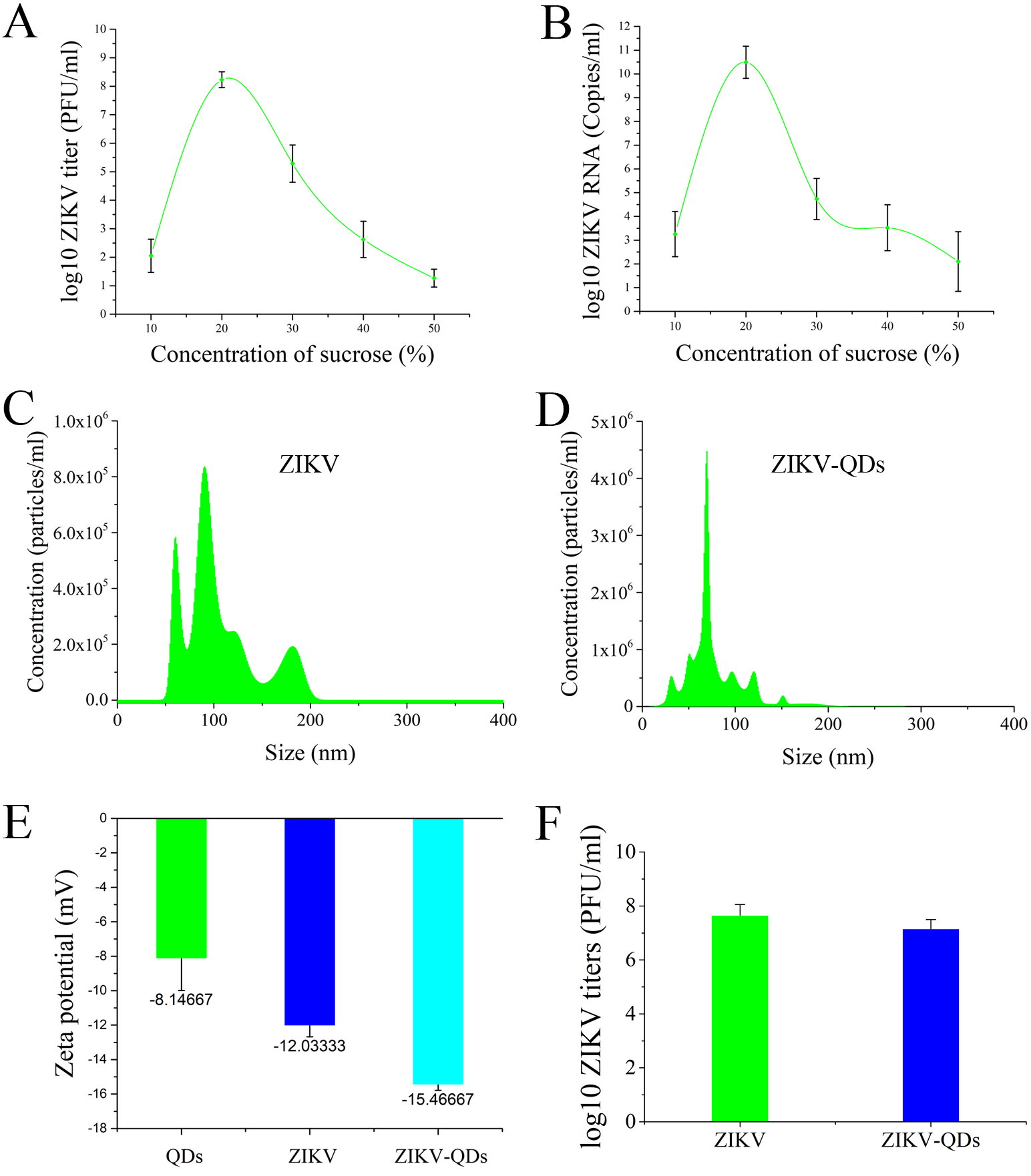


Figure S7. The particle sizes of QDs.


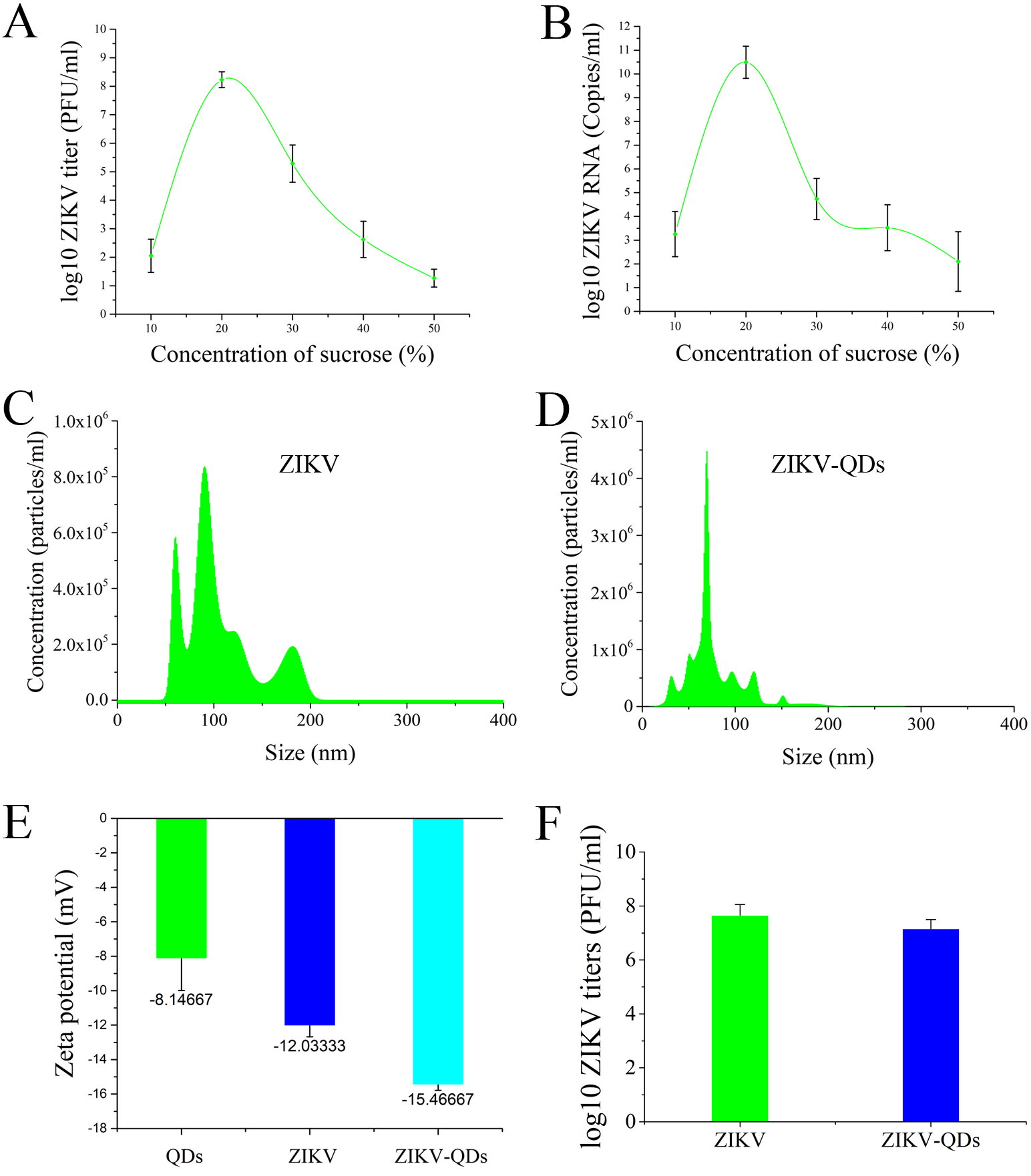


Figure S8. The particle sizes of ZIKV-QDs.


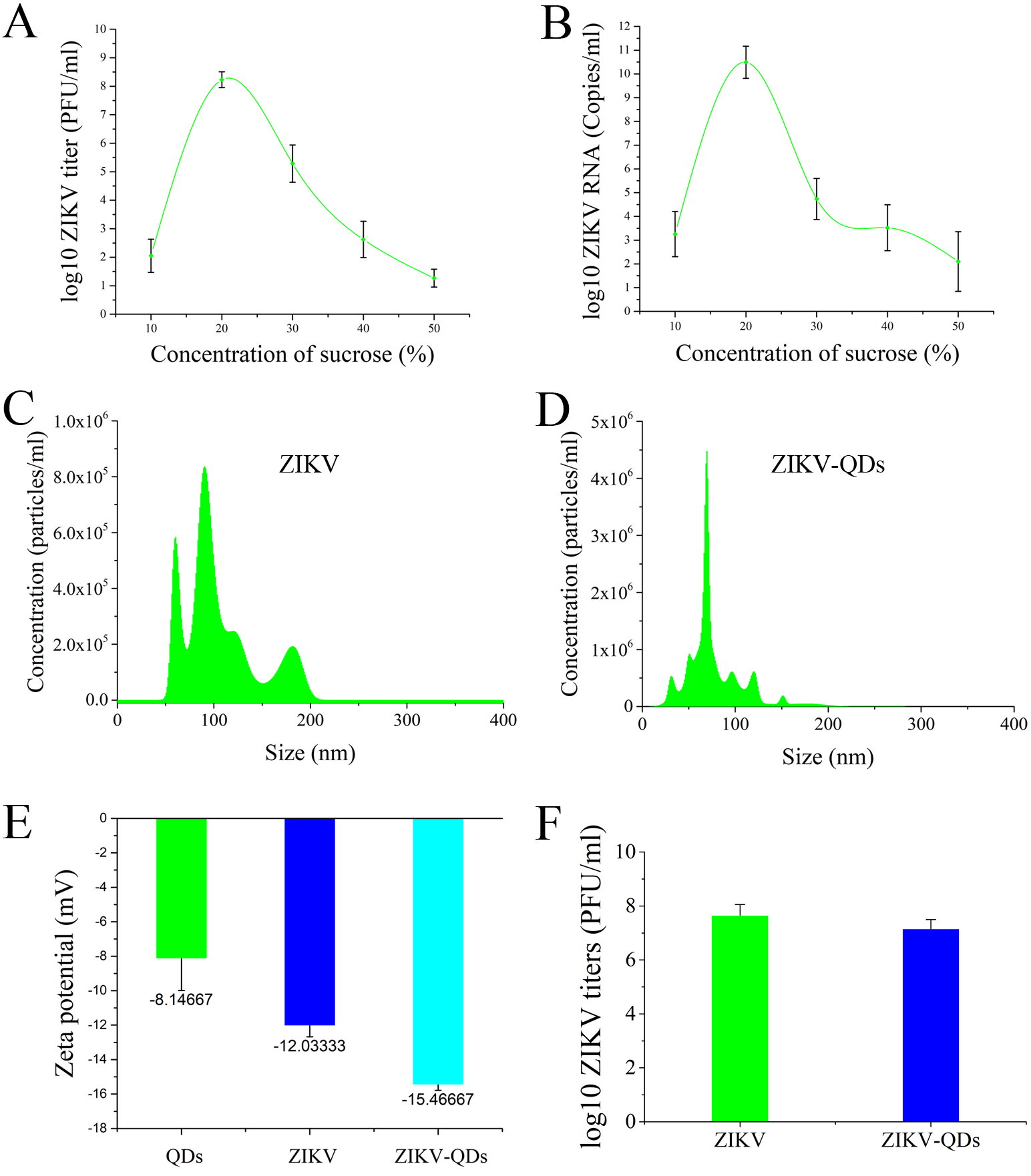


Figure S9. The zeta potential data of the complex of QDs, ZIKV and ZIKV-QDs


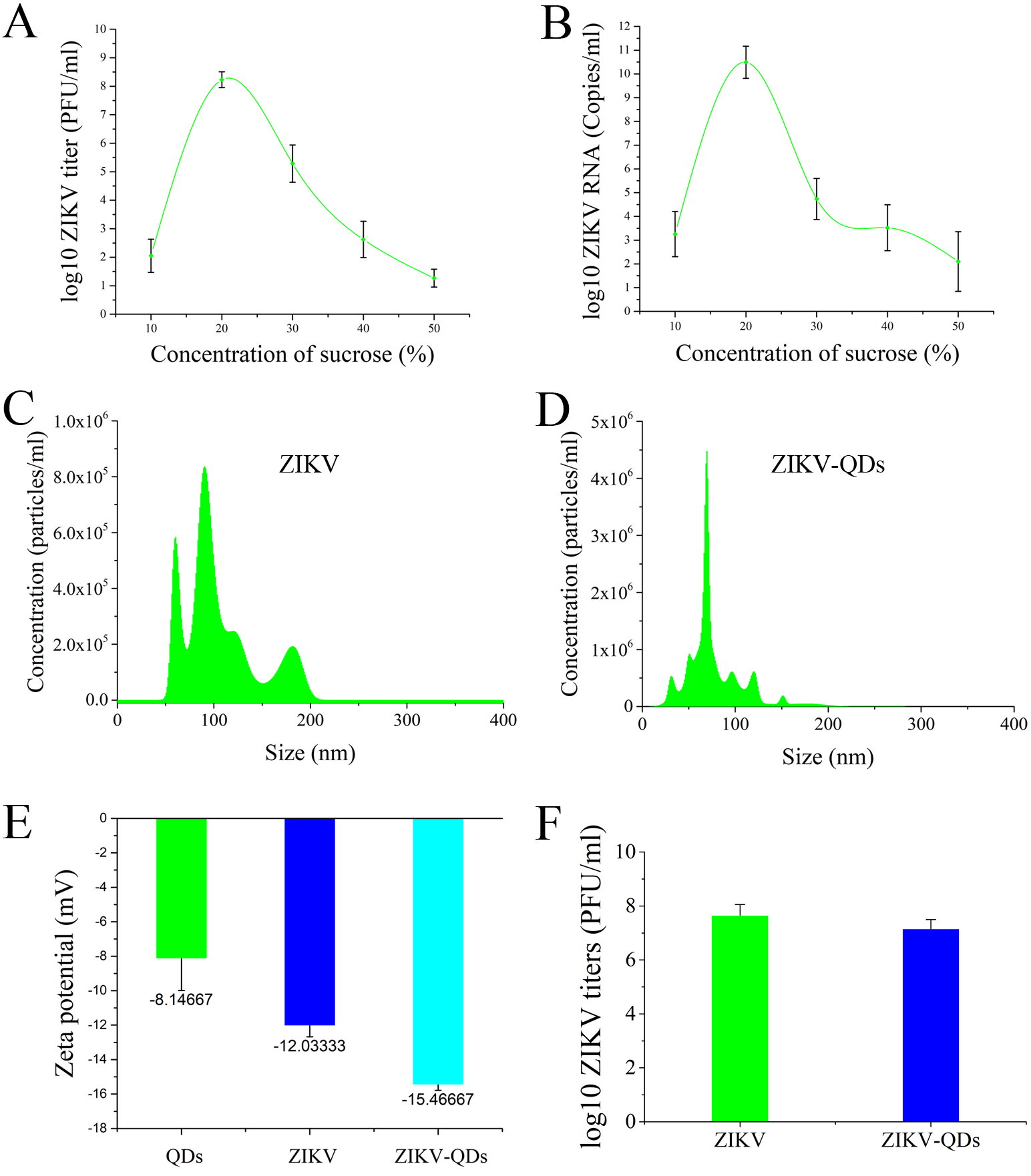


Figure S10. The titers counting of the purified fraction of ZIKV and ZIKV-QDs virus.


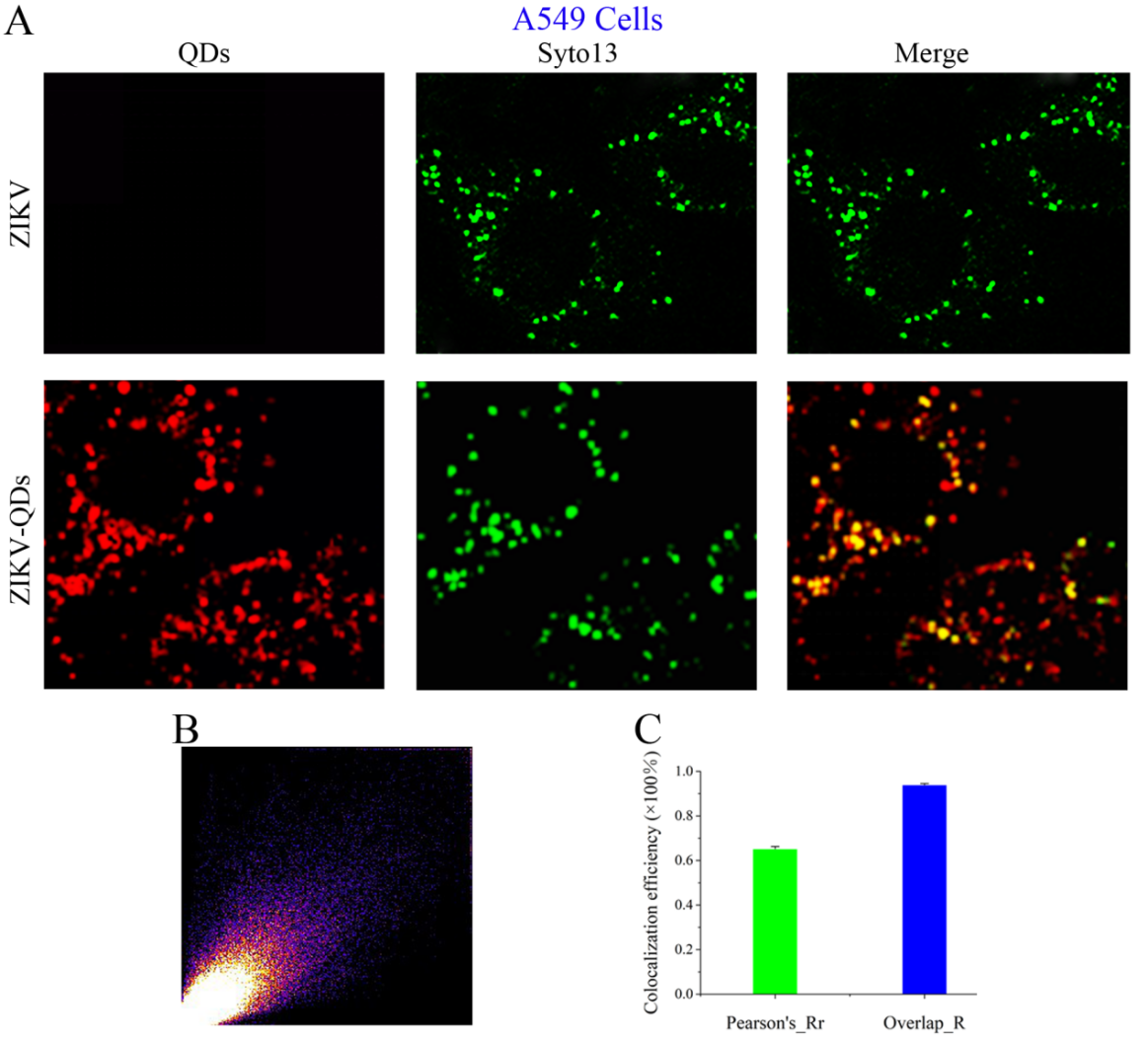


Figure S11. (A) Confocal microscopic images of ZIKV nucleic acid colocalized with the commercially available organelle trackers Syto13 in A549 cells. (B) Colocalization scatterplots of (A). (C) the corresponding Pearson Correlation coefficient of (A).
